# Hedgehog Inhibition Promotes a Switch from Type II to Type I Cell Death Receptor Signaling in Cancer Cells

**DOI:** 10.1371/journal.pone.0018330

**Published:** 2011-03-31

**Authors:** Satoshi Kurita, Justin L. Mott, Sophie C. Cazanave, Christian D. Fingas, Maria E. Guicciardi, Steve F. Bronk, Lewis R. Roberts, Martin E. Fernandez-Zapico, Gregory J. Gores

**Affiliations:** 1 Division of Gastroenterology and Hepatology, Mayo Clinic, Rochester, Minnesota, United States of America; 2 Division of Oncology Research, Schulze Center for Novel Therapeutics, Mayo Clinic, Rochester, Minnesota, United States of America; University of Texas MD Anderson Cancer Center, United States of America

## Abstract

TRAIL is a promising therapeutic agent for human malignancies. TRAIL often requires mitochondrial dysfunction, referred to as the Type II death receptor pathway, to promote cytotoxicity. However, numerous malignant cells are TRAIL resistant due to inhibition of this mitochondrial pathway. Using cholangiocarcinoma cells as a model of TRAIL resistance, we found that Hedgehog signaling blockade sensitized these cancer cells to TRAIL cytotoxicity independent of mitochondrial dysfunction, referred to as Type I death receptor signaling. This switch in TRAIL requirement from Type II to Type I death receptor signaling was demonstrated by the lack of functional dependence on Bid/Bim and Bax/Bak, proapoptotic components of the mitochondrial pathway. Hedgehog signaling modulated expression of X-linked inhibitor of apoptosis (XIAP), which serves to repress the Type I death receptor pathway. siRNA targeted knockdown of *XIAP* mimics sensitization to mitochondria-independent TRAIL killing achieved by Hedgehog inhibition. Regulation of XIAP expression by Hedgehog signaling is mediated by the glioma-associated oncogene 2 (GLI2), a downstream transcription factor of Hedgehog. In conclusion, these data provide additional mechanisms modulating cell death by TRAIL and suggest Hedgehog inhibition as a therapeutic approach for TRAIL-resistant neoplasms.

## Introduction

Tumor necrosis factor–related apoptosis-inducing ligand (TRAIL), a potent death ligand that preferentially induces apoptosis in transformed cells, initiates signaling cascades by ligating two death receptors, DR4 (TNFRSF10A, also referred to as TRAIL receptor 1) and DR5 (TNFRSF10B, also referred to as TRAIL receptor 2/KILLER/TRICK-2) [1]. TRAIL-induced clustering and oligomerization of DR4 and DR5 results in conformational changes of the death domains within their cytoplasmic tails, facilitating recruitment and activation of caspases 8 and 10 within a death inducing signaling complex (DISC) [2], [3], [4]. If activation of these initiator caspases is sufficiently robust, they directly activate caspase 3, which in turn results in cellular demise by the so-called Type I death receptor pathway of apoptosis [5], [6]. However, if the magnitude of caspase 8 and 10 activation is not sufficient to directly activate caspase 3, TRAIL may still induce apoptosis through cleavage of the proapoptotic BH3-only protein Bid to generate truncated Bid (tBID). tBid protein triggers mitochondrial outer membrane permeabilization by promoting oligomerization of the pro-apoptotic Bcl-2 family proteins Bak and/or Bax within this membrane. Mitochondrial outer membrane permeabilization results in egress of pro-apoptotic proteins from the mitochondrial intermembrane space (i.e., cytochrome c, Smac/DIABLO, HtrA2, AIF, and endonuclease G), which culminates in the mitochondrial pathway of apoptosis [7], [8]; the dependence on mitochondrial dysfunction for cell death is referred as the Type II death receptor pathway of apoptosis [6]. Type I death receptor signaling appears to be regulated at the level of caspase-3 activation, whereas Type II death receptor signaling is regulated at the level of mitochondria by members of the antiapoptotic Bcl-2 protein family [7], [8], [9], [10]. TRAIL usually signals through the Type II, mitochondria-dependent pathway [11], a pathway which is frequently inhibited in cancers resulting in TRAIL resistance [12], [13].

Herein, we demonstrate that inhibition of the oncogenic Hedgehog pathway represses the expression of XIAP and sensitizes cancer cells to TRAIL cytotoxicity, using cholangiocarcinoma cells as a model to study TRAIL resistance. These cancer cells abundantly express antiapoptotic Bcl-2 family proteins, especially Mcl-1, which mediates TRAIL resistance [14], [15]. Mcl-1 inhibition of the mitochondrial pathway of cell death is especially relevant to cholangiocarcinoma cells as they paradoxically express TRAIL *in vivo*, and likely must continuously circumvent TRAIL cytotoxic signaling for survival [16]. Given the emerging data implicating oncogenic Hedgehog signaling in gastrointestinal tumor biology [17], [18], we explored Hedgehog signaling as a mechanism contributing to TRAIL resistance by cancer cells. We previously reported that the Hedgehog pathway is constitutively active in these cells and that its inhibition sensitizes cholangiocarcinoma cells to TRAIL cytotoxicity coinciding with up-regulation of TRAIL receptor DR4 [19]. However, it was unclear how up-regulation of DR4 alone could overcome the Mcl-1 blockade of the mitochondrial pathway of apoptosis. In this study, we have demonstrated that down-regulation of XIAP by Hedgehog inhibition converts Type II TRAIL signaling to a Type I pathway. Thus, our data define a mechanism by which Hedgehog signaling modulates TRAIL-induced cell death in cancer cells and suggest inhibition of this cascade as potential therapeutic approach for TRAIL-resistant neoplasms..

## Materials and Methods

### Ethics Statement

The Mayo Clinic IRB Committee reviewed and approved for human studies the the protocol entitled “Genome expression analysis of human cholangiocarcinoma (CCC).” The committee determined that this constitutes minimal risk research. Data were analyzed anonymously.

### Cell Culture

The human cholangiocarcinoma cell lines, KMCH, HuCCT-1, and Mz-ChA-1, were cultured in Dulbecco's modified Eagle's medium (DMEM) supplemented with 10% fetal bovine serum, 100,000 units/L penicillin, 100 mg/L streptomycin and 100 mg/L gentamicin as previously described [20], [21].

### Quantitative reverse-transcription polymerase chain reaction (RT-PCR)

Total RNA was isolated from cells using TRIzol reagent (Invitrogen, Carlsbad, CA), and cDNA was prepared using random primers and Moloney murine leukemia virus reverse transcriptase as previously described in detail [22]. The cDNA product was amplified by PCR with Taq DNA polymerase using standard protocols. PCR primers are depicted in Table 1. Primers for 18S ribosomal RNA (rRNA) were purchased from Ambion Inc. (Austin, TX). Real-time PCR was performed with the Roche LightCycler using SYBR green as the fluorophore as previously described [20]. The copy number of the target mRNA in each sample was normalized as a ratio using the copy number for 18S rRNA in the denominator.

**Table 1:** Sequences for real-time PCR primers.

Gene	Primer pair
cIAP-1	5′-AGCTAGTCTGGGATCCACCTC-3′
	5′-GGGGTTAGTCCTCGATGAAG-3′
cIAP-2	5′-TGGAAGCTACCTCTCAGCCTAC-3′
	5′-GGAACTTCTCATCAAGGCAGA-3′
XIAP	5′-GATGCTGTGAGTTCTGATAGG-3′
	5′-CTTAATGTCCTTGAAACTGAAC-3′

### Immunoblot Analysis

Whole-cell lysates were obtained as previously described by us in detail [21]. Protein samples were resolved by SDS-PAGE gels, transferred to nitrocellulose membranes, and blotted with the indicated primary antibodies at a dilution of 1:500 to 1:1,000. Peroxidase-conjugated secondary antibodies (Biosource International, Camarillo, CA) were incubated at a dilution of 1:3,000 to 1:5,000. Bound antibodies were visualized using enhanced chemiluminescence reagents (Amersham, Arlington Heights, IL) and Kodak X-OMAT film (Eastman Kodak, Rochester, NY). Primary antisera were those raised to actin, Bcl-2, Bcl-x, Mcl-1, and Smoothened (Santa Cruz Biotechnology, Santa Cruz, CA: C11, B19, S18, S19, and H-300, respectively), Bid and cIAP-1 (R&D Systems, Minneapolis, MN), Bim and XIAP (BD Biosciences, San Jose, CA), and c-IAP2 (Novus Biologicals, Littleton, CO). For quantitation of XIAP protein by immunoblot, films were analyzed by densitometry and the relative signal intensity of the XIAP signal was normalized to actin in three separate experiments.

### Apoptosis

Apoptosis was quantified morphologically by staining the nuclei with 4′,6-diamino-2-phenylindole dihydrochloride (DAPI). The characteristic nuclear changes of apoptosis (*i.e*. chromatin condensation and nuclear fragmentation) were assessed by fluorescence microscopy using excitation and emission wavelengths of 380 and 430 nm, respectively. Caspase-3/7 activity in cell cultures was employed to assess apoptosis biochemically and was measured by quantifying fluorophore release from the caspase-3/7 substrate using the Apo-ONE^TM^ homogeneous caspase-3/7 kit (Promega, Madison, WI).

### RNA interference

A specific double-stranded 21-nucleotide RNA sequence complementary to the target message was used to silence human cIAP-1, XIAP, Bak or Bax expression. Validated siRNAs targeting cIAP-1 or XIAP were purchased from Santa Cruz Biotechnology. siRNAs targeting Bak or Bax were designed and synthesized using the software available at www.ambion.com and the Silencer siRNA Construction kit from Ambion (Austin, TX). The siRNA sequences employed to inhibit Bak or Bax expression were as follows: *Bak,* 5′-AAG TAC GAA GAT TCT TCA AAT **CCT GTC TC**-3′; *Bax,* 5′-AAG ACG AAC TGG ACA GTA ACA **CCT GTC TC**-3′ (partial T7-promoter sequence is bold). As a control, cells were transfected with a scrambled RNA duplex with the following sequence: 5′-AAC GTG ATT TAT GTC ACC AGA-3′. Briefly, cells grown in 6-well dishes were transiently transfected with 30 nM siRNA using 5 µL/mL siPORT^TM^ NeoFX^TM^ (Ambion Inc.). Target protein expression was assessed by immunoblot analysis after transfection with the siRNA.

Short hairpin RNA (shRNA) for Bid was from Sigma Aldrich [MISSION short hairpin RNA lentiviral plasmid, targeting Nucleotides 376-396, Genebank accession #NM_001196]. To generate the Bim targeted shRNA construct, the pSSH1 plasmid containing the human H1 promoter for the expression of shRNA was employed. A double-stranded DNA template (5′-GAT CCC C**GC AAT AGG CTT TAG GAA AA**T TCA AGA GA**T TTT CCT AAA GCC TAT TGC** TTT TTG GAA A-3′) was inserted into the pSSH1 plasmid after the H1 RNA promoter. The DNA insert contains sense and antisense sequences (Bold type) for the Bim mRNA and a 9-nucleotide linker sequence, yielding transcription of a shRNA targeting Bim. For stable transfection, KMCH cells were transfected using OPTI-MEM I (GIBCO-Invitrogen, Carlsbad, CA) containing 5 µL/mL LipofectAMINE 2000 (Invitrogen), and 3 µg/mL plasmid DNA. Forty-eight hours after transfection, fresh medium containing 2 µg/mL puromycin was added for shRNA plasmid targeting Bid or 1.2 g/L neomycin was added for shRNA plasmid targeting Bim. Surviving clones were separated using cloning rings and were individually cultured. Lentiviral particles containing shRNA to SMO were constructed using the BLOCK-iT Inducible H1 Lentiviral RNAi System (Invitrogen), following the manufacturer's protocol. Sequences (inserted into pLenti4/BLOCK-iT-DEST) were depicted as previously described by us [19]. For stable transfection, fresh medium containing 10 µg/mL Blasticidine-S and 500 µg/mL Zeocin (Invitrogen) was added to cells 48 hours after transduction. Surviving clones were separated using cloning rings and were individually cultured. Expression was induced with 1 mg/ml tetracycline (Sigma-Aldrich, St. Louis, MO). After an additional 48 hours cells were used for experiments. Target suppression was assessed by immunoblot analysis.

### Electrophoretic mobility shift assay (EMSA)

Nuclear cell extracts were prepared using NE-PER nuclear and cytoplasmic extraction reagents (Pierce, Rockford, IL) according to the manufacturer's instructions. For the EMSA, 20 µg of nuclear proteins were incubated at room temperature for 20 min in binding buffer (25 mM HEPES, pH 7.5, 0.1 M NaCl, 2 mM EDTA, 6% glycerol, 0.1% Triton X-100, 0.1 mM phenylmethylsulfonyl fluoride, 0.4 mM dithiothreitol, 0.5 µg/µL poly(dI-dC), 0.5 µg/µL salmon sperm) with 0.04 pmol of Cy5.5-labeled double-stranded DNA oligonucleotide (synthesized by the Mayo Clinic Advanced Genomics Technology Core, Rochester MN) containing the wild-type putative GLI binding sequences within the promoter region of human *XIAP* gene. Protein-DNA complexes were separated from the unbound DNA probe by electrophoresis through 5% native polyacrylamide gels containing 0.5X Tris borate-EDTA. Fluorescence was visualized directly on the gel using an Odyssey fluorescent imager (Licor Biosciences, Lincoln, NE). For competition assays, a 200-fold molar excess of unlabeled double-stranded oligonucleotide was added to the reaction mixture 20 min before the addition of the fluorescent probe.

### Chromatin Immunoprecipitation

ChIP was performed from KMCH cells treated with 250 nM recombinant Sonic Hedgehog for 5 hours using total cellular DNA sheared to ∼500 bp fragments, using ExactaCHIP chromatin immunoprecipitation kit (R&D systems) following manufacturer's instructions and samples were pre-cleared using agarose beads plus salmon sperm slurry (Upstate, Lake Placid, NY) prior to immunoprecipitation. Positive control primers supplied by the manufacturer were for the *Bcl-2* promoter (bound by GLI1 and GLI2) and the *GLI1* promoter (bound by GLI3). Primers for *XIAP* promoter site I were, Forw: 5′ TTACTGCAGCCTCCAACTCC; Rev: 5′ CATCTCTCCATGCTCTGAACTC.

### Statistical Analysis

All data represent at least three independent experiments and are expressed as mean ± SE. Differences between groups were compared using ANOVA with Bonferroni post hoc correction.

### Materials

Reagents were purchased from the following suppliers: DAPI was from Sigma; recombinant human TRAIL and recombinant Sonic Hedgehog were from R&D Systems; anti-human Fas antibody (CH-11) was from MBL International (Nagoya, Japan); cyclopamine was from LC Laboratories (Woburn, MA).

## Results

### Smoothened inhibition down-regulates cIAP-1 and XIAP expression

**Figure 1:**
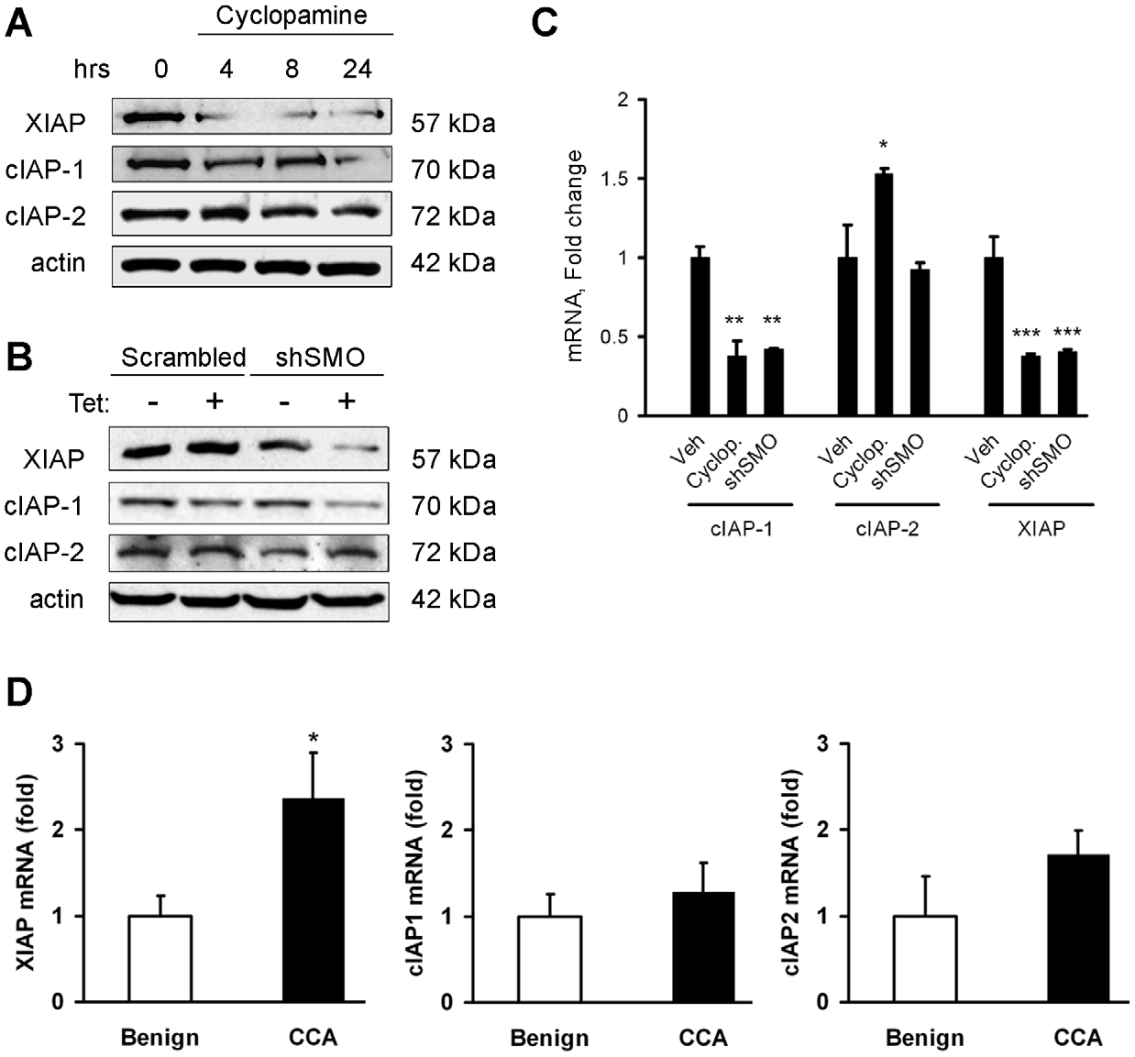
Smoothened inhibition down-regulates both cIAP-1 and XIAP expression. *A,* Cell lysates from KMCH cells treated with cyclopamine (5 µM) for the indicated times were immunoblotted for cIAP-1, cIAP-2 and XIAP. Actin was used as a loading control. *B,* KMCH cells were transfected with a tetracycline-inducible shRNA expression vector targeting Smoothened (shSMO) or a scrambled shRNA vector; shRNA expression was induced for 48 hours by treatment with tetracycline (1 mg/mL) followed by immunoblotting for cIAP-1, cIAP-2 and XIAP using whole cell lysates. *C*, Cells were treated with vehicle, cyclopamine (5 µM) for 24 hours, or transfected with shSMO as in panel *B,* then analyzed by real-time RT-PCR for *cIAP-1*, *cIAP-2* and *XIAP* using total RNA. *D*, Total RNA from tissue samples of either cholangiocarcinoma (CCA) or adjacent benign liver was used to assess *XIAP, cIAP-1, and cIAP-2* mRNA expression, normalized to 18S and expressed as fold change from benign. Mean ± SEM, *p<0.05; **p<0.01; ***p<0.001 compared to vehicle.

Given a pivotal mechanistic role for inhibitor of apoptosis proteins (IAPs) in death receptor signaling [23] and the role Hedgehog in cellular survival, we first ascertained if inhibiting Smoothened (SMO), the signaling component of the Hedgehog receptor complex, modulates expression of IAPs. Treatment with cyclopamine, a well-established pharmacological inhibitor of SMO [24], [25], decreased cellular IAP-1 (cIAP-1) and X-linked IAP (XIAP) protein levels, but had no effect on cIAP-2 protein expression. The down-regulation of XIAP was more rapid, occurring within 4 hours, whereas loss of cIAP-1 protein occurred only after 24 hours (Fig. 1A). The pharmacological effect of cyclopamine was validated by using shRNA targeted knockdown of SMO, a signaling component of the Hedgehog receptor, which also decreased both cIAP-1 and XIAP cellular protein levels, but not cIAP-2 (Fig. 1B). To determine if SMO inhibition affected mRNA levels or acted exclusively post-transcriptionally, *cIAP-1*, *cIAP-2*, and *XIAP* mRNA expression was determined after cyclopamine treatment or shRNA to SMO. Both *XIAP* and *cIAP-1* mRNA levels were decreased by SMO inhibition (Fig. 1C). The clinical relevance of IAP regulation was assessed in tumor and adjacent benign samples from patients with cholangiocarcinoma. A two-fold increase in *XIAP* mRNA levels was observed in tumor tissue (Fig. 1D). The levels of *cIAP-1* and *-2* were not significantly different. That *cIAP-1* is not elevated in tumor samples despite regulation by Hedgehog (Fig. 1C) may indicate additional as-yet-unknown factors also contribute to control of IAP expression in the tumor. These data suggest the Hedgehog signaling pathway regulates *cIAP-1* and *XIAP* expression, but that only *XIAP* levels are elevated in clinical samples. Therefore, we largely focused on *XIAP* for the remainder of our studies.

### GLI2 binds to the *XIAP* promoter and regulates its expression

**Figure 2:**
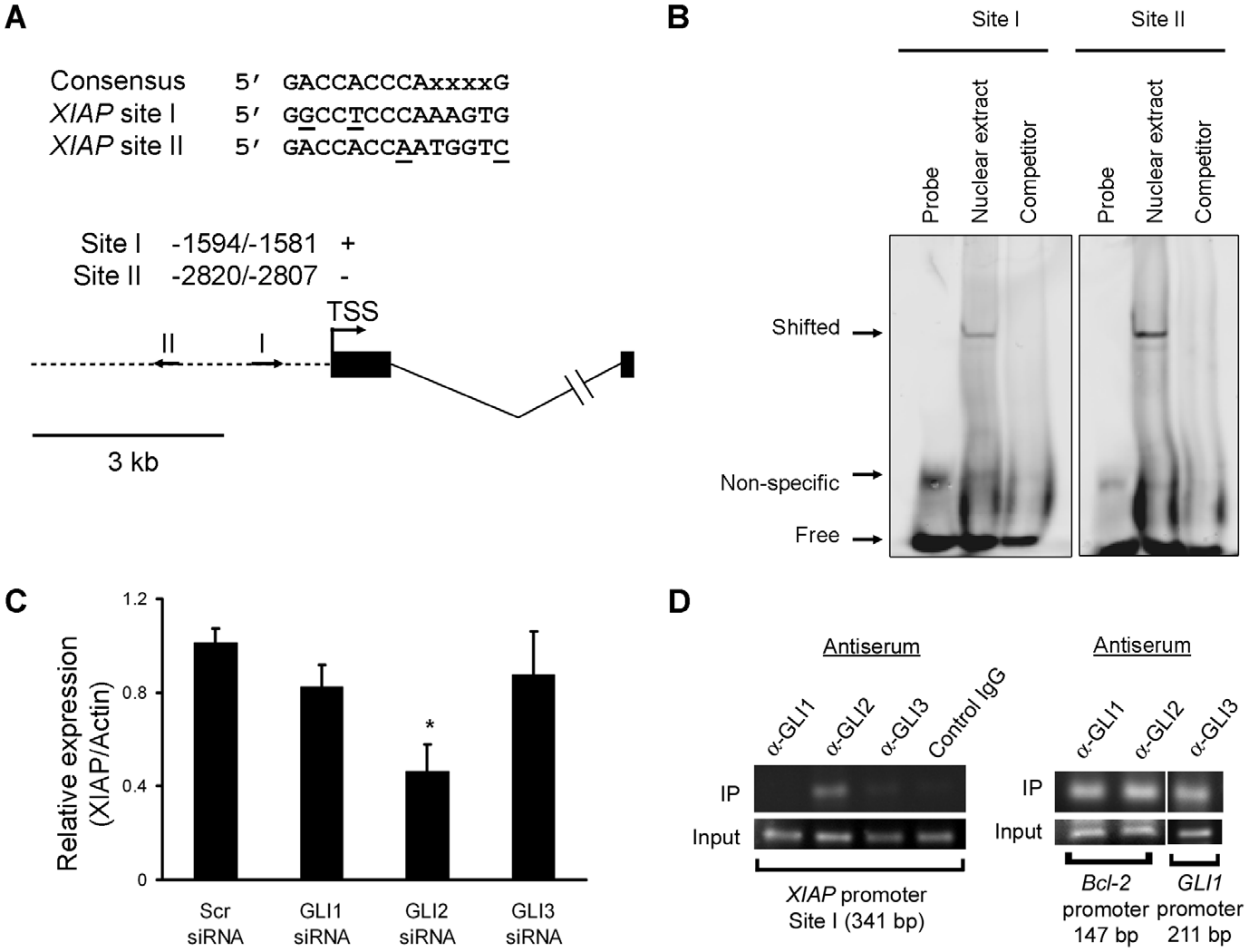
XIAP promoter predicted GLI-binding sites exhibit sequence-specific binding to nuclear proteins. *A,* Putative GLI binding sites in the XIAP promoter region. Nucleotide positions were counted from the transcription start site (TSS). Potential binding sites were observed as indicated by arrows on the schematic (I and II). *B,* Nuclear protein extracts were isolated from KMCH cells. EMSA was performed using Cy5.5-labeled double-stranded oligonucleotides containing the putative GLI binding sequences within the human XIAP promoter, and competition experiments with 200-fold molar excess cold oligonucleotides as described under “MATERIALS AND METHODS”. *C*, Total cellular protein was isolated 48 hours after transient transfection with the indicated siRNA against GLI family members. XIAP protein expression was determined by immunoblotting and the signal was quantified by densitometry as a ratio to actin expression. *D,* Chromatin immunoprecipitation using antiserum to GLI1, GLI2, GLI3, or a control IgG was performed followed by PCR using primers flanking site I (341 bp) within the *XIAP* promoter region, as indicated. As a positive control, ChIP was performed using primers flanking the *Bcl-2* promoter GLI-binding site (GLI1 and GLI2), or primers flanking the GLI3 binding site within the *GLI1* promoter.

To determine if *XIAP* is transcriptionally regulated by Hedgehog-responsive GLI transcription factors, we searched the 5 Kb 5′-flanking region of the human *XIAP* gene for the 5′- *GACCACCCAXXXXG* -3′ GLI consensus binding motif. Two promising candidate GLI-binding sites (Fig. 2A) were identified upstream of the *XIAP* transcription start site (NM_001167) at positions −2820/−2807 (Site I) and −1594/−1581 (Site II). Each had two mismatches as compared to the consensus sequence [26]; Site I is identical to the validated 9-nucleotide Bcl-2 GLI-binding site, Bs1 [27], while Site II is identical to the confirmed DR4 GLI-binding site [19]. We performed electrophoretic mobility shift assay experiments. Binding to both putative GLI binding sequences was observed in nuclear extracts from KMCH cells. The mobility shift of CY 5.5-labeled probe could be prevented by adding a 200-fold molar excess of the unlabeled probe (Competitor; Fig. 2B). Of the three GLI family members, siRNA to GLI2 caused a significant decrease in XIAP protein expression while siRNA to GLI1 or GLI3 did not alter XIAP levels (Fig. 2C). To ascertain if GLIs bind to the *XIAP* promoter, we performed chromatin immunoprecipitation experiments to assess occupation of the endogenous *XIAP* promoter using antibodies to GLI1, GLI2, and GLI3. Binding of GLI2 was observed using primers that amplify Site I (Fig. 2D). Control IgG did not yield a product, demonstrating specificity of the antisera used, and positive-control promoter sites demonstrated the expected occupation by GLI1 and GLI2 (*Bcl-2* promoter), and GLI3 (*GLI1* promoter). These data suggest that Hedgehog directly regulates *XIAP* expression by GLI2 transcription factor binding to Site I in the *XIAP* promoter.

### Sensitization by Hedgehog inhibition is independent of mitochondrial apoptotic mediators, Bid/Bim or Bax/Bak

**Figure 3:**
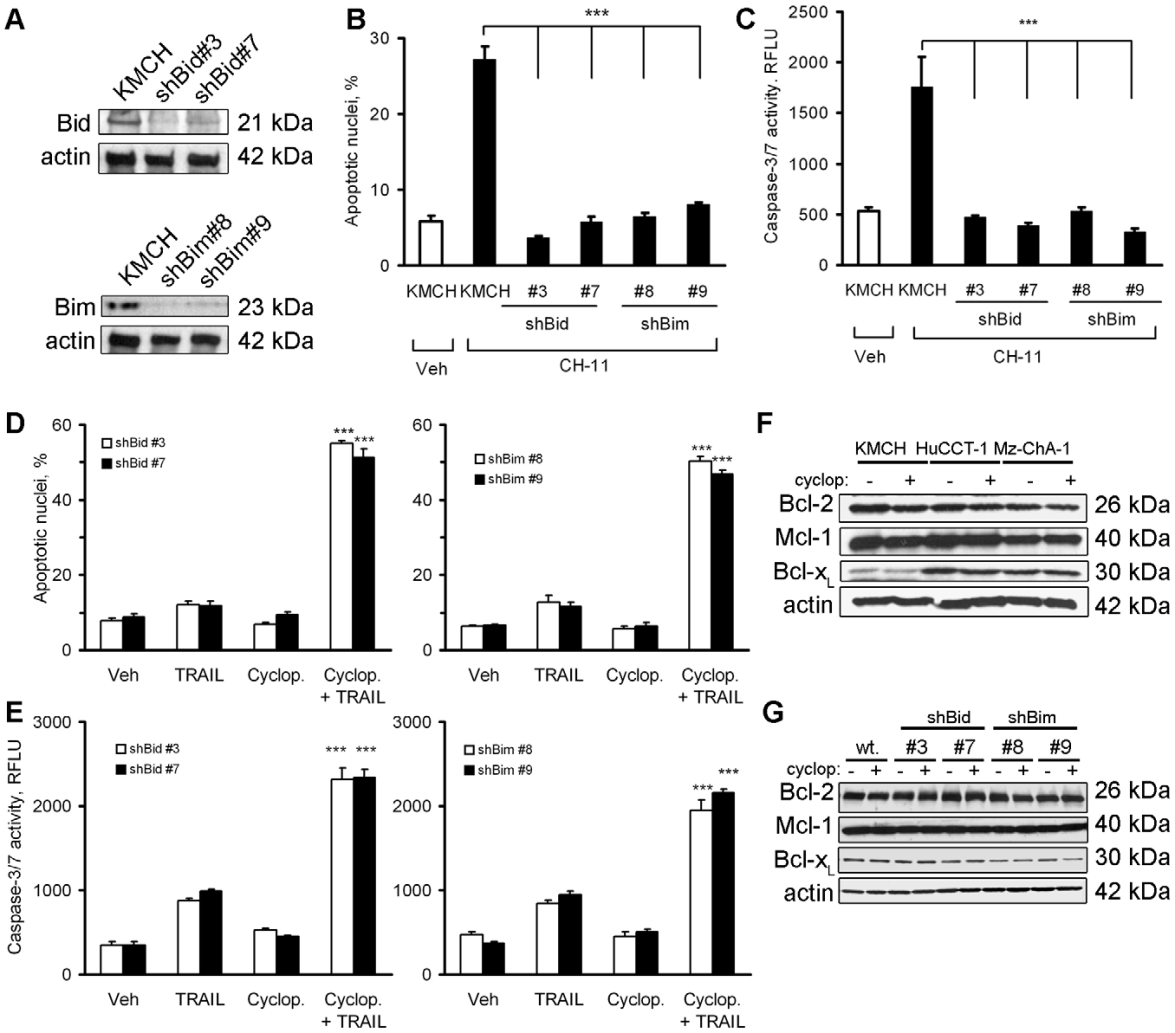
Cyclopamine sensitizes cells to TRAIL-induced cell death despite knockdown of Bid or Bim. *A,* Immunoblot analysis was performed on whole cell lysates obtained from KMCH cells stably transfected with the specific shRNA targeting *Bid* or *Bim,* using indicated antisera. Actin was used as a loading control. *B*, KMCH cells stably transfected with Bid- or Bim-shRNA and untransfected parental KMCH cells were treated with Fas agonistic antibody CH-11 (100 µg/mL) for 5 hours followed by DAPI staining. Cells with apoptotic morphology were counted. Mean ± SEM, ***p<0.001 compared to parental cells treated with CH-11. *C,* In parallel, caspase-3/7 activity was measured in cells treated as in panel *B*. Mean ± SEM, ***p<0.001. *D*, KMCH cells stably transfected with Bid- or Bim-shRNA were pretreated with vehicle or cyclopamine (5 µM) for 24 hours, and then treated with TRAIL (5 ng/mL) for 5 hours followed by DAPI staining. Cells with apoptotic morphology were counted. Mean ± SEM, ***p<0.001 compared to cells treated with TRAIL alone. *E*, KMCH stable cell lines were treated as in panel *D*, and after 5 hours caspase-3/7 activity was measured. Mean ± SEM, ***p<0.001 compared to cells treated with TRAIL alone. *F*, Whole cell lysates from KMCH, HuCCT-1, and Mz-ChA-1 cells treated with vehicle or cyclopamine (5 µM) for 24 hours were analyzed by immunoblot using anti-Bcl-2, Mcl-1, or Bcl-x antisera. Actin was used as a loading control. *G*, Whole cell lysates were obtained from shBid-, shBim- and untransfected parental KMCH cells treated with vehicle or cyclopamine (5 µM) for 24 hours and were analyzed by immunoblot using anti-Bcl-2, Mcl-1, or Bcl-x antisera. Actin was used as a loading control.

XIAP is a key discriminator for converting Type II to Type I death receptor signaling [9], thus, we considered loss of XIAP protein as a mechanism by which Hedgehog inhibition sensitized cholangiocarcinoma cells to TRAIL-induced apoptosis. Bid and Bim are key signaling intermediates in Type II proapoptotic death receptor signaling [28], [29]. Based on this information, we next ascertained if Hedgehog inhibition sensitized cells to TRAIL cytotoxicity in the absence of Bid or Bim. shRNA constructs targeting Bid and Bim efficiently knocked down the respective cellular protein levels (Fig. 3A). The near complete silencing of Bid or Bim protein levels was functionally confirmed by taking advantage of the observation that Fas Type II death receptor signaling is dependent upon Bid or Bim [30]. Indeed, Bid or Bim knockdown was sufficient to inhibit Fas-induced cell death by the agonistic antibody CH-11 (Fig. 3B and 3C), a classic Type II pathway of cell death [31]. Thus, if TRAIL-induced cell death in cyclopamine-sensitized cells was dependent on Type II signaling, then Bid or Bim silencing should similarly protect from TRAIL cytotoxicity. However, despite the absence of Bid or Bim, the Hedgehog inhibitor cyclopamine still sensitized human cholangiocarcinoma cells to TRAIL-induced cell death (Fig. 3D and 3E).

Because an imbalance between anti- and proapoptotic Bcl-2 family proteins by cyclopamine could explain its effects on sensitizing cells to TRAIL killing, we profiled this class of proteins by immunoblot analysis. Bcl-x_L_ protein expression was significantly lower in KMCH cells than HuCCT-1 or Mz-ChA-1 cells, but none of the three antiapoptotic proteins (Bcl-2, Mcl-1, or Bcl-x_L_) was altered by cyclopamine treatment (Fig. 3F). Cyclopamine also did not alter cellular protein levels of the antiapoptotic Bcl-2 family proteins in KMCH cells stably transfected with shRNA targeting Bid or Bim (Fig. 3G). Likewise, SMO inhibition does not alter BH3-only proteins or pro-apoptotic Bcl-2 family proteins, Bax and Bak [19]. Taken together, these data suggest that inhibition of the Hedgehog pathway sensitizes human cholangiocarcinoma cells to TRAIL cytotoxicity by a process independent of Bid or Bim or alterations in the expression of multidomain Bcl-2 family proteins. The independence of Bid or Bim suggested the intriguing possibility that cyclopamine may sensitize cholangiocarcinoma cells to TRAIL by converting apoptotic signaling from a Type II to a Type I death receptor pathway.

**Figure 4:**
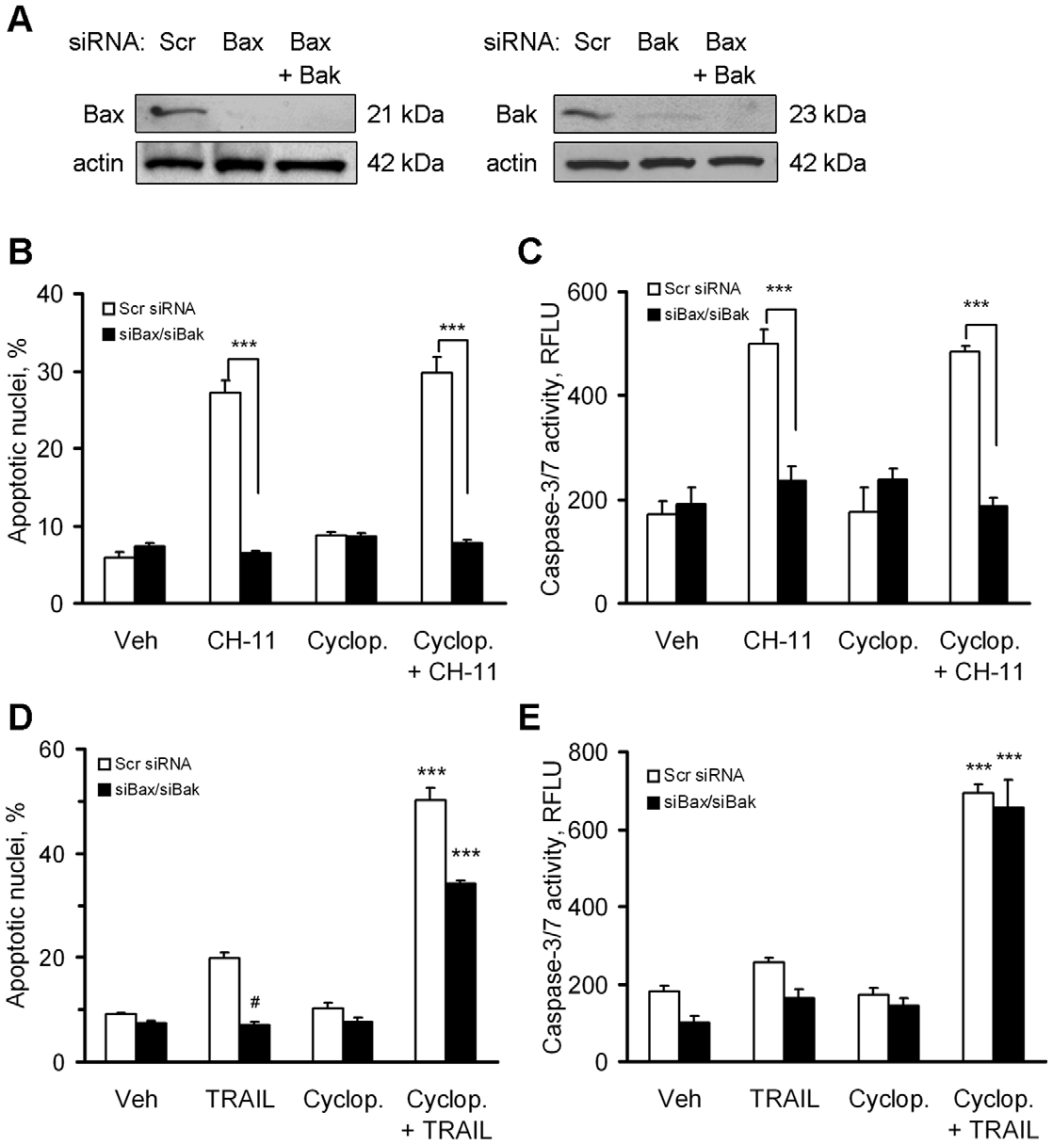
Cyclopamine sensitizes cells to TRAIL-induced cell death independent of Bax and Bak. *A,* Whole cell lysates from KMCH cells transiently transfected with specific siRNA targeting Bax and/or Bak or scrambled siRNA were obtained 48 hours after transfection and analyzed by immunoblot using anti-Bax or anti-Bak antisera. Actin was used as a loading control. *B*, KMCH cells transiently transfected with Bax- and Bak-siRNA or scrambled siRNA were pretreated (24 hours) with medium or cyclopamine (5 µM) as indicated, then treated with Fas agonistic antibody CH-11 (100 µg/mL) for 5 hours. Cells with apoptotic nuclear morphology were counted. Mean +/− SEM; ***p<0.001. *C,* KMCH cells were treated as in panel *B* and caspase-3/7 activity was measured after 5 hours. Mean +/− SEM; ***p<0.001 *D*, KMCH cells transiently transfected with Bax- and Bak-siRNA or scrambled siRNA were pretreated with vehicle or cyclopamine (5 µM) for 24 hours, and then treated with TRAIL (5 ng/mL) for 5 hours. Cells with apoptotic nuclear morphology were counted. Mean ± SEM, #p<0.05 and ***p<0.001 compared to cells transfected with scrambled siRNA and treated with TRAIL. *E*, KMCH cells transiently transfected with indicated siRNA and treated as in panel *D*, were assayed after 5 hours for caspase-3/7 activity. Mean ± SEM; ***p<0.001.

To test this hypothesis, we next determined whether Hedgehog inhibition converts Type II death receptor signaling to Type I signaling. The sine qua non of Type I death receptor signaling is its lack of dependence on Bax and Bak [32]. Efficient knockdown of Bax and Bak protein levels was achieved by siRNA targeting (Fig. 4A). Targeted knockdown of Bax plus Bak protein functioned to inhibit Fas-mediated apoptosis by CH-11 (Fig. 4B and 4C), consistent with Type II signaling. TRAIL alone had a small apoptotic effect on KMCH cells that was efficiently blocked by Bax/Bak silencing, indicating that TRAIL normally kills cholangiocarcinoma cells by the Type II pathway (Fig. 4D). However, TRAIL-induced apoptosis was dramatically increased by cyclopamine (Fig. 4D and 4E). The increased apoptosis was completely (Fig. 4E) or nearly completely (Fig. 4D) insensitive to Bax/Bak silencing. These data suggest that Hedgehog inhibition circumvents mitochondrial resistance to TRAIL cytotoxicity by converting a Type II signaling to a Type I signaling pathway.

### Knockdown of *XIAP* sensitized cholangiocarcinoma cells to TRAIL-induced apoptosis despite inhibition of Bid

**Figure 5:**
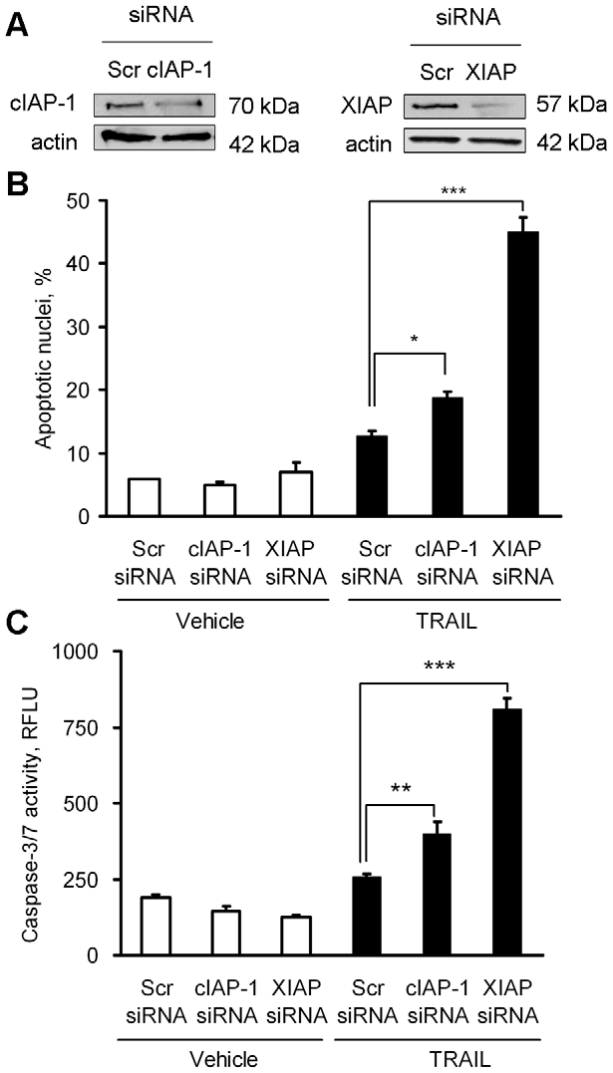
Knockdown of XIAP sensitizes human cholangiocarcinoma cells to TRAIL cytotoxicity. KMCH cells were transfected with scrambled siRNA or specific siRNA for *cIAP-1* or *XIAP*. *A*, Whole cell lysates were prepared for immunoblotting from KMCH cells 48 hours after siRNA transfection. *B*, Forty-eight hours after transfection as in panel *A*, cells were treated with human recombinant TRAIL (5 ng/mL) or medium for 5 hours. Cells were then stained with DAPI and cells with apoptotic nuclear morphology were counted and expressed as a percentage of total nuclei. *C,* In parallel, cells were transfected and treated with TRAIL as in panel *B*, and after 5 hours caspase-3/7 activity was measured. Mean ± SEM, *p<0.05; **p<0.01; ***p<0.001.

Next, we determined the functional relevance of XIAP expression in these cancer cells using siRNA-targeted knockdown of *cIAP-1* or *XIAP.* Efficient targeting of cIAP-1 and XIAP protein expression was confirmed by immunoblot analysis (Fig. 5A). Consistent with previous reports [33], [34], [35], [36], knockdown of *XIAP* significantly sensitized the cells to TRAIL cytotoxicity, while knockdown of *cIAP-1* only modestly sensitized the cells to TRAIL-induced cell death, measured either morphologically (Fig. 5B) or biochemically (Fig. 5C). Thus, regulation of XIAP expression is a potential mechanism by which Hedgehog signaling prevents TRAIL cytotoxicity.

**Figure 6:**
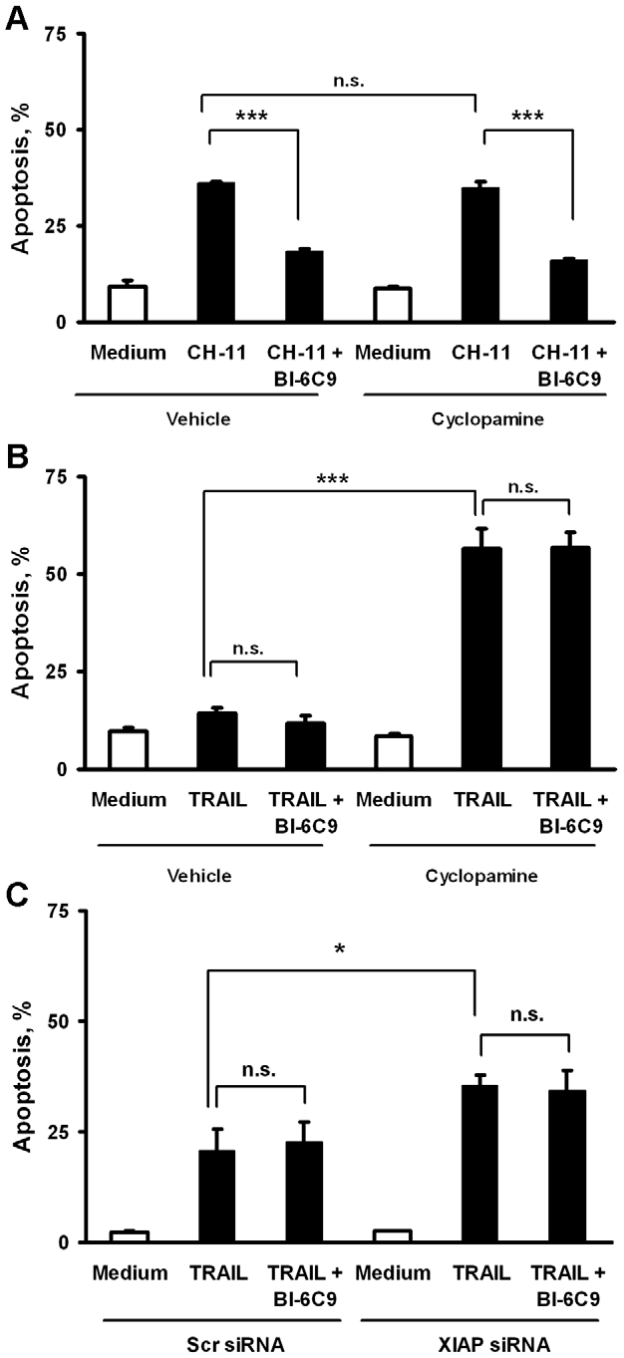
XIAP knockdown sensitizes cells to TRAIL-induced cell death despite Bid inhibition. *A,* KMCH cells were incubated for 24 hours in medium or cyclopamine (5 µM) followed by treatment with either vehicle (DMSO, final concentration 0.1% v/v) or the Bid inhibitor, BI-6C9 (10 µM) for 1 hour. Cells were subsequently treated with Fas agonistic antibody CH-11 (100 µg/mL) for an additional 5 hours (where appropriate, BI-6C9 remained in the medium during CH-11 treatment). Cells with apoptotic nuclear morphology were quantified as referenced in the Materials and Methods. Mean +/− SEM; ***p<0.001; n.s. indicates p>0.10 by ANOVA with Bonferroni correction. *B,* KMCH cells were incubated overnight with or without cyclopamine followed by BI-6C9 as in panel *A*, except that apoptosis was induced by TRAIL (5 ng/mL) for 5 hours (where appropriate, BI-6C9 remained in the medium during TRAIL treatment) and apoptotic nuclear morphology was quantified. Mean +/− SEM; ***p<0.001; n.s. indicates p>0.10 by ANOVA with Bonferroni correction. *C,* KMCH cells transiently transfected with XIAP siRNA or scrambled siRNA (48 hours) were pretreated with BI-6C9 (10 µM) or DMSO vehicle (final concentration 0.1% v/v) for 1 hour. Next, the cells were treated with TRAIL (5 ng/mL) for 5 hours (where appropriate, BI6C9 remained in the medium during TRAIL treatment). Cells with apoptotic nuclear morphology were quantified. Mean ± SEM, *p<0.05; n.s. indicates p>0.10 by ANOVA with Bonferroni correction.

The above data are consistent with a switch from Type II to Type I receptor-mediated apoptosis by SMO inhibition; SMO inhibition appears to mediate this switch by down-regulating *XIAP* gene expression. To test this interpretation of the data, we designed experiments to ascertain if knockdown of XIAP protein was sufficient to convert TRAIL signaling to a mitochondria-independent, Type I pathway. We employed a small molecule inhibitor of Bid, BI-6C9, to circumvent mitochondria-dependent cell death [37]. First, to confirm that BI-6C9 functionally inhibited cell death in this system, KMCH cells were pre-treated with BI-6C9 or vehicle (DMSO), followed by Fas agonistic antibody. Indeed, pharmacologic inhibition of Bid by BI-6C9 was sufficient to repress Fas-induced apoptosis. Again, SMO inhibition did not affect apoptosis by CH-11 treatment (Fig. 6A). In contrast, BI-6C9 did not functionally inhibit TRAIL-induced cell death in cyclopamine-treated cells (Fig. 6B). Importantly, siRNA directed against XIAP also sensitized cells to TRAIL-induced apoptosis, and BI-6C9 did not mitigate cell death (Fig. 6C). Thus, SMO inhibition, by decreasing cellular XIAP protein levels, converts Type II TRAIL death receptor signaling to a Type I pathway; an effect recapitulated by siRNA targeted knockdown of *XIAP*.

## Discussion

Results of the present study provide new insights into the mechanisms by which the Hedgehog pathway inhibits TRAIL cytotoxicity. The results demonstrate that in human cholangiocarcinoma cells, (i) Hedgehog pathway inhibition sensitizes human cholangiocarcinoma cells to TRAIL-mediated apoptosis, in part, by down-regulating XIAP protein; (ii) *XIAP* repression by SMO inhibition occurs at the mRNA level as well, consistent with GLI-mediated enhancement of *XIAP* transcription; and (iii) inhibition of Hedgehog promotes TRAIL cytotoxicity independent of Bid, Bim, Bax, and Bak. These data suggest inhibition of Hedgehog signaling modulates TRAIL cytotoxicity in human cholangiocarcinoma cells by regulating XIAP expression and converting TRAIL signaling from Type II to Type I apoptotic signaling. Each of these results is discussed in greater detail below.

Hedgehog signaling components are constitutively expressed by cholangiocarcinoma cells [19], [38]. Indeed, this pathway is active in human cholangiocarcinoma cell lines in an autocrine and/or paracrine manner as cyclopamine suppresses the growth of cholangiocarcinoma cells both *in vitro* and *in vivo* [38] and imparts survival signals [19]. Our current study extends these observations by suggesting Hedgehog oncogenic signaling regulates a non-mitochondrial pathway of apoptosis. Indeed, despite inhibiting TRAIL cytotoxicity, Hedgehog does not appear to alter expression of antiapoptotic Bcl-2 family proteins which govern the mitochondrial cell death pathway. Instead, Hedgehog appears to suppress expression of the TRAIL receptor DR4 [19] and promotes expression of XIAP, which work in concert to reduce TRAIL cytotoxicity.

**Figure 7:**
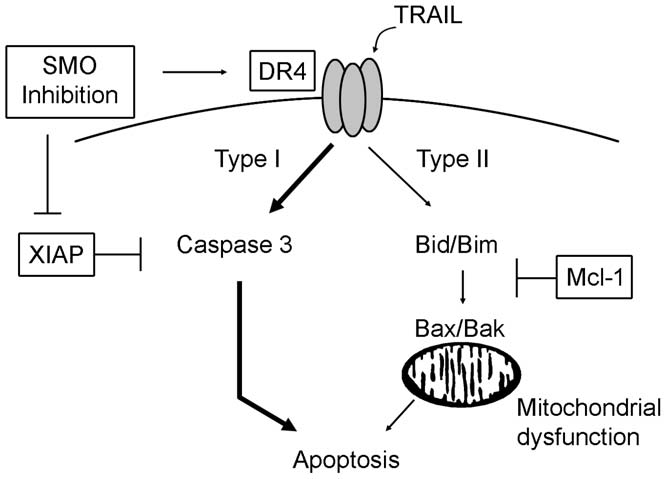
SMO inhibition sensitizes cells to TRAIL-induced apoptosis by increasing DR4 and decreasing XIAP protein levels, thereby switching cells from Type II to Type I death receptor signaling. Schematic illustration of the interaction of Hedgehog and TRAIL signaling in cholangiocarcinoma cells. Death signaling upon TRAIL binding to DR4 normally is dependent on mitochondrial dysfunction (induced by Bid/Bim as well as Bak/Bax activation) to induce cholangiocarcinoma cell death (Type II pathway), a pathway blocked by elevated Mcl-1 protein levels in cholangiocarcinoma cells. Alternately, following DR4 ligation, robust caspase 3 activation can induce Type I death signaling, a pathway inhibited by XIAP expression under normal conditions. Inhibition of SMO expression or activity sensitizes cells to TRAIL via increasing DR4 [19] and decreasing XIAP levels, thereby switching cells from Type II to Type I death receptor signaling and promoting apoptosis.

Cholangiocarcinoma cells express robust amounts of antiapoptotic Bcl-2 family proteins, in particular Mcl-1, which mitigates TRAIL cytotoxicity [14], [15], consistent with a mitochondria-dependent, Type II death receptor signal. In contrast, we found that SMO inhibition sensitized cholangiocarcinoma cells to TRAIL even after depletion of Bid, Bim, Bax, or Bak by specific siRNA, suggesting mitochondrial dysfunction is not required for TRAIL killing in this model. Finally, genetic inhibition of XIAP not only sensitized cholangiocarcinoma cells to TRAIL killing, but did so independently of mitochondrial involvement (i.e. in the presence or absence of Bid inhibition). The degree of sensitization to TRAIL by XIAP siRNA was less than that seen by SMO inhibition (c.f. Fig. 6B and 6C), indicating that cyclopamine likely affects additional targets to fully sensitize cells to apoptosis. One plausible candidate is the TRAIL receptor DR4 [19]. Recent reports suggest XIAP may have a similar role in pancreatic cancer, as XIAP inhibition similarly restored TRAIL sensitivity by converting cytotoxic signaling from a Type II to a Type I death receptor pathway in several TRAIL-resistant cancer cell lines [33], [34], [35]. Our investigation confirms that XIAP inhibition can switch to mitochondria-independent death receptor signaling and integrates this observation into the protective effects of constitutive Hedgehog signaling observed in cholangiocarcinoma cells. A schematic illustration of the interaction of TRAIL signaling and SMO inhibition is presented in Figure 7. Based on the results of the current study, as well as current concepts in death receptor signaling, we propose that blocking constitutive Hedgehog signaling via SMO inhibitors increases DR4 mRNA and protein levels [19] as well as inhibiting XIAP expression. This combined action sensitizes cells to TRAIL killing by the Type I death receptor pathway, bypassing the Mcl-1 mediated block in Type II TRAIL killing.

Of note, cyclopamine did not convert Fas signaling to a Type I pathway, suggesting differential signaling pathways for these two death receptors. We speculate that Fas signaling and TRAIL signaling are affected differently by Hedgehog inhibition. Specifically, cholangiocarcinoma cells are sensitive to Fas-agonist induced killing at baseline in contrast to TRAIL, and while cyclopamine sensitized cells to TRAIL killing, it did not alter Fas-agonist sensitivity. Further, Hedgehog inhibition did not alter Fas protein expression, but increased DR4 at both the mRNA and protein levels [19]. Potentially, the combined effects of decreasing cellular XIAP protein levels plus enhancing DR4 expression by Hedgehog inhibition may explain this differential regulation of death ligand signaling.

In conclusion, our study implicates Hedgehog stimulation of XIAP expression as a mechanism for TRAIL resistance in human cholangiocarcinoma cells. As these cancers express TRAIL *in vivo*, pharmacologic inhibition of Hedgehog signaling may promote autocrine and/or paracrine cholangiocarcinoma-autonomous cytotoxicity. Collectively, these concepts suggest Hedgehog pathway inhibitors, which are currently in clinical development [39], [40], may have therapeutic efficacy in the treatment of human cholangiocarcinoma.
